# Hemodynamic Response in Ascending Aorta Surgery Patients during Moderate Intensity Resistance Training

**DOI:** 10.1155/2023/7616007

**Published:** 2023-10-28

**Authors:** Rikke Gottlieb, Kasper Arnskov, Marius Henriksen, Eva Prescott, Hanne Rasmusen, Christian Have Dall

**Affiliations:** ^1^Department of Physical Therapy and Occupational Therapy, Copenhagen University Hospital, Bispebjerg-Frederiksberg Hospital, Copenhagen, Denmark; ^2^Department of Cardiology, Copenhagen University Hospital, Bispebjerg-Frederiksberg Hospital, Copenhagen, Denmark; ^3^Department of Occupational Therapy and Physical Therapy, Copenhagen University Hospital, Rigshospitalet, Copenhagen, Denmark; ^4^The Parker Institute, Copenhagen University Hospital, Bispebjerg-Frederiksberg Hospital, Copenhagen, Denmark

## Abstract

**Background:**

In patients undergoing ascending aortic surgery (AAS), postsurgical physical exercise with a safe and effective exercise prescription is recommended. Resistance training is associated with blood pressure (BP) elevations that may increase the risk of new aortic dissection or rupture. However, the acute hemodynamic response to resistance training for this patient group is unknown.

**Aim:**

The aim of this study was to investigate peak systolic BP (SBP) increases in AAS patients during moderate intensity resistance training.

**Methods:**

SBP was measured continuously beat-to-beat with a noninvasive method during three sets of leg presses at moderate intensity. A 15-repetition maximum strength test was performed to estimate the maximal amount of resistance a participant could manage 15 times consecutively (equivalent to approximately 60–65% of their maximum strength).

**Results:**

The study had 48 participants in total, i.e., 24 cases and 24 controls. Both groups consisted of 10 females (42%) and 14 males (58%). The case group had a mean age of 60.0 (SD ± 11.9) years and a mean of 16.3 months since surgery (minimum 4.4 and maximum 39.6 months). 22 of the 24 cases received antihypertensive medication. The median baseline BP was 119/74 mmHg among cases and 120/73 mmHg among controls. During the first set of leg presses, the median peak SBP was 152 mmHg, in the second set 154 mmHg, and in the third set 165 mmHg. Corresponding values in controls were 170 mmHg, 181 mmHg, and 179 mmHg. The highest peak SBP registered in an AAS patient was 190 mmHg and in any healthy control was 287 mmHg.

**Conclusion:**

The findings indicate that AAS patients in control of their BP have the endurance to perform 3 sets of resistance training at moderate intensity as their SBP increases with a maximum of 39% from the baseline compared to the 51% increase in the control group.

## 1. Introduction

Degenerative, genetical, and congenital conditions all predispose to aortic aneurysms, dilatations, dissection, or rupture which results in elective or urgent surgery [1, 2]. These conditions are associated with high mortality and are primarily treated with ascending aortic surgery (AAS) where the aortic tissue is replaced with a graft [2, 3]. With increasing ageing of the population and improvement in surgical methods, an increasing number of patients undergo AAS [4–6].

Resting blood pressure is measured following ascending aortic surgery, with a goal of <120/70 mmHg, and is achieved through medical treatment. The patients are monitored closely until 120/70 mmHg is reached [1, 7].

The level of acute rise in blood pressure following ascending aortic surgery is not documented, and there does not exist a documented limit for blood pressure increases during physical activity, e.g., a brisk walk for this patient group. Despite a paucity of evidence, it is presumed that sudden acute elevations in blood pressure (as occur in heavy weightlifting) may increase the risk of a new aortic dissection or rupture [8]. Because of this assumption and due to safety, a general recommendation for the upper limit of acute blood pressure increases in the patient group is lacking and physical activity recommendations and restrictions are opinion and experience-based and not supported by clinical evidence or studies of safety [8]. A lifestyle survey revealed that the number of patients not engaged in any structured physical activity increased after postaortic dissection due to fear of a new dissection [8].

Training-based rehabilitation of patients with coronary heart disease (CHD) reduces cardiovascular mortality and improves physical capacity and health-related quality of life [9, 10].

An observational pilot study shows that some forms of cardiac rehabilitation are safe and helpful for the Marfan syndrome patients who have undergone AAS or heart valve surgery [11]. Resistance training was not offered in the training-based rehabilitation in this study.

AAS patients are recommended to avoid isometric exercises and be physically active with a safe and effective exercise prescription to prevent conditions associated with a physically inactive lifestyle such as diabetes and arteriosclerosis [8, 12, 13].

Research indicates a lower BP postresistance training and a positive effect on age-related loss of balance, muscle strength, and mass [14–17]. Guidelines from the European Society of Cardiology contain recommendations for cardiac fitness and resistance training in training-based rehabilitation [9, 18, 19]. The recommendations for CHF patients NYHA class II-III are either low intensity (40–50% of max strength) or moderate intensity (60% of max strength) [9, 18, 19]. These guidelines were used to guide AAS patients in Denmark as there were no specific recommendations for AAS patients other than to avoid isometric resistance training at the time this study was conducted [7, 18, 20].

A few studies have addressed the acute effect of endurance training on peak SBP in AAS patients. In a study of 26 AAS patients doing moderate intensity cycling, 75% of the patients had a SBP between 150 and 170 mmHg and the other 25% had a SBP <150 mmHg (50). In a study of 29 AAS patients, maximum SBP was measured during a VO_2_ peak test before and after exercise-based rehabilitation vs. no training [21]. Mean maximum SBP was 207 ± 33 mmHg. A third study has addressed the hemodynamic responses in AAS patients during cardiopulmonary exercise testing (*n* = 128) [22]. No serious adverse events were observed, and peak exercise systolic and diastolic blood pressures were 160/70 mmHg.

In a systematic literature search, no studies were identified in the field of AAS patients' acute SBP increase during resistance training.

Therefore, the overall aim of this current study was to investigate the peak systolic BP in AAS patients during moderate intensity resistance training (leg press) according to current European guidelines. Second, the study aimed to compare peak systolic BP in AAS patients with that in a healthy gender and age-matched control group to evaluate the differences between the groups.

## 2. Methods

### 2.1. Design

A descriptive single-center intervention study with 24 AAS cases and 24 healthy controls.

### 2.2. Participants

Participants who had undergone AAS were recruited from a cardiac rehabilitation unit in Copenhagen and fulfilled all the inclusion criteria (adult AAS patients (+18 years), >eight weeks postoperatively, approved for training by a cardiologist, able to perform a leg-press exercise, and able to understand instructions in Danish or English) and none of the exclusion criteria (medically treated B-dissection, resting SBP >150 mmHg, or left ventricular ejection fraction (LVEF) <15% assessed with ultrasound by a cardiologist). All the participants had participated in a rehabilitation programme at the time when the study was conducted.

The participants in the healthy control group fulfilled all of the inclusion criteria (same sex and age as the matched case participant (±5 years), able to perform a leg-press exercise, and able to understand instructions in Danish or English) and none of the exclusion criteria (any heart disease or resting SBP >150 mmHg).

### 2.3. Hemodynamic Outcomes

Continuous beat-to-beat measurements of the hemodynamics in real time were acquired by a Nexfin monitor (BMEYE, Amsterdam, Netherlands). The accuracy of the method is investigated in various reports and is comparable with intra-arterial measurements with a difference in systolic pressure measurements but not of a clinical relevance [23–27].

Participants had a finger clamp applied on the middle finger between the proximal interphalanges joint and the distal interphalanges joint, which kept the artery at constant volume by applying counter pressure [24].

### 2.4. Study Procedure

Participants attended one test session at the cardiac rehabilitation unit in Copenhagen. Resting BP was measured after lying down for five minutes in a quiet room. Thereafter, the participants did a 15-minute warmup on a bicycle ergometer, keeping a constant speed of 60–80 rpm. After the warmup, they were positioned in a Technogym leg-press machine “leg-press horizontal/seated mechanical” with their feet placed in a parallel position.

**Figure 1:**
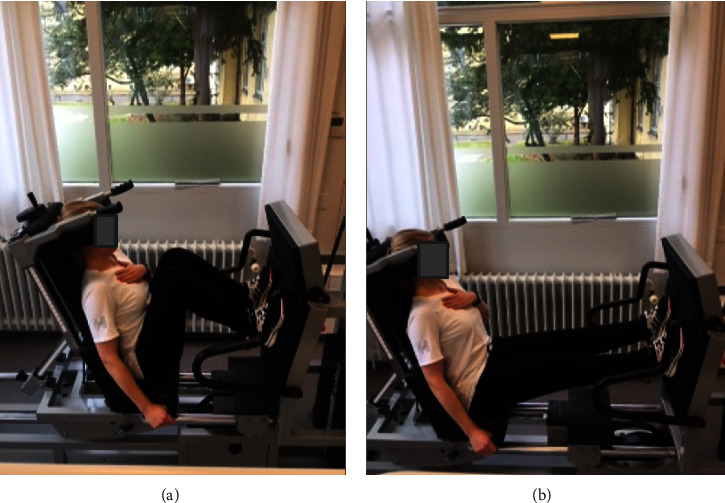
Positioning and movement in the leg-press device. (a) Legs bended and no resistance. (b) Legs extended and resistance lifted. Including continuously beat-to-beat SBP measurements on the left hand situated on the participants' chest during the whole exercise.

A 15-repetition maximum (RM) strength test was performed to estimate the maximal amount of resistance a participant could manage 15 times consecutively (equivalent to approximately 60–65% of their maximum strength) [19]. Once the correct resistance was determined, the participant had a finger clamp applied on the middle finger of their left hand to indirectly measure the BP continuously and noninvasively. The participants were instructed to place their left hand on their chest and their right hand on the right handle of the machine and to hold this position during the entire examination (Figure 1). Furthermore, the participants were instructed to refrain from speaking, to move nothing but their legs, and avoid squeezing or moving their hands during the entire examination.

Each participant performed three sets of 15 repetitions (each set lasting approximately 70 seconds), and 60 seconds rest periods were given in between sets to ensure adequate recovery time for participants [19, 28, 29]. As a safety precaution, instructions in the correct breathing technique during resistance training were provided; participants were asked to exhale during the most strenuous phase and inhale during the less strenuous phase of each repetition. The participants were furthermore asked to avoid Valsalva Manoevre (forced exhalation against a closed glottis) which could lead to further systolic BP increases [23, 29, 30]. Furthermore, there was a pragmatically safety set maximum at 200 mmHg which means that the entire examination stops if the SBP reached 200 mmHg in the case group.

### 2.5. Statistical Analysis

**Figure 2:**
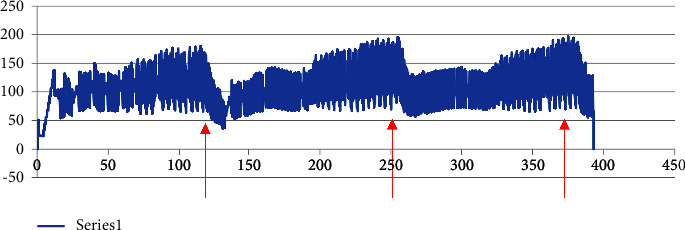
Example of a continuous blood pressure measurement during three sets of leg press. *Y*-axis: systolic BP (mmHg) and *X*-axis: seconds. Figure 2 shows an example of a continuous dataset with three sets of leg press including breaks. The three red arrows indicate the three peak systolic BP's, and these are the data intervals used for further analysis.

Data were extracted from the Nexfin monitor in beat-to-beat data (data points for each heartbeat) and continuous data (Figure 2). Beat-to-beat data were used for further analysis. Continuous data were used to validate the beat-to-beat data to avoid using artifacts/outliers in the analysis and to avoid using data from calibrating points.

To use the correct data points from the, respectively, large dataset collected from the 48 participants, a set of macros were coded collecting the data and combining them into a single file with mean, minimum, and maximum values for the baseline and each of the peaks from the three sets. For each exercise set, the highest systolic and diastolic BP, HR, and workload were selected for further analysis.

Analyses were conducted using the SPSS statistics IBM (Version 24). Data are reported as the median, mean values, +standard deviation (SD), 95% confidence intervals and range, or frequency percentages. For comparison of continuous variables, unpaired Student's *t*-test was applied. For nominal variables, an *X*^2^-test was conducted. To investigate if age, months since operation, or amount of antihypertensive prescriptions impacted SBP increase, a linear regression analysis with Pearson correlation (*r*) was performed. Model assumptions were checked by visual inspection of residual plots. The threshold for a statistical significance was set at *p* < 0.05.

### 2.6. Ethical Approval

The Danish Data Protection Agency approved the study (j.nr.: 2012-58-0004). The Health Research Committee in the Capital Region deemed the study exempt from approval (H-17041675). The study was registered at https://clinicaltrials.gov/ before commencement of any study-related activities (NCT03424863) and was conducted in accordance with the World Medical Association Declaration of Helsinki.

Subjects were informed orally and in writing about the study and signed informed consent afterwards.

## 3. Results

**Figure 3:**
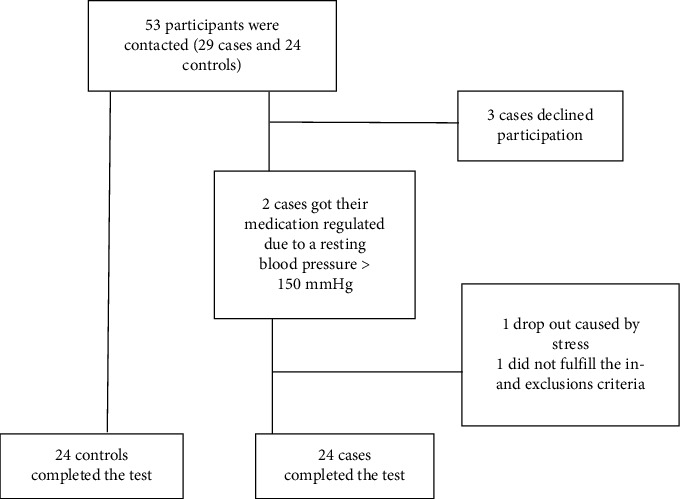
Flow of participants through the study. Three possible participants declined participation, one possible participant dropped out before the intervention due to stress, and one possible participant did not fulfil the in- and exclusion criteria.

The study had 48 participants in total: 24 in the AAS group and 24 in the control group (flowchart Figure 3). Both groups consisted of 10 females (42%) and 14 males (58%). The case group had a mean age of 60.0 (SD ± 11.9) years and the control group 60.2 (SD 11.8) years. The participants in the AAS group had a mean of 16.3 months since surgery (minimum 4.4 and maximum 39.6 months).

22 of the AAS patients received antihypertensive treatment. In the control group, 3 of the participants received the lowest possible dose of antihypertensive treatment. Resting SBP was significantly lower in patients than in controls (121 ± 12.9 versus 128 ± 11.8, *P*=0.042) whereas resting HR and BMI were higher. All characteristics of the two groups are shown in Table 1.

Among the control group, only four participants used any type of medication, i.e., angiotensin II antagonist (*n* = 2), statins (*n* = 1), and calcium antagonists (*n* = 2). In the AAS group, three participants only had the use of anticoagulants whereas everyone else used two or more medications, a total of 98 prescriptions. Most common prescriptions were anticoagulants (*n* = 20), beta-blocks (*n* = 14), statins (*n* = 10), diuretics (*n* = 8), angiotensin II antagonist (*n* = 7), ACE-inhibitors (*n* = 7), and calcium antagonists (*n* = 9).

Beta-blockers, angiotensin II antagonist, ACE-inhibitors, calcium antagonists, and diuretics are all antihypertensive drugs. In the case group, 31% (*n* = 7) were treated with only one antihypertensive drug, 31% (*n* = 7) were treated with two drugs combined, and 31% (*n* = 7) were treated with three or more drugs combined. The control group had 4 participants (*n* = 16%) with comorbidities (as Alzheimer and arthrosis) versus the AAS group with only four participants (*n* = 16%) without comorbidities. The comorbidities in the AAS group were diabetes, COPD, asthma, and arrhythmia.

### 3.1. Hemodynamic Responses to Leg Press in AAS

The hemodynamics before and during the three sets of leg-presses are shown in Table 2.

In the AAS group, the baseline SBP increased by 34 mmHg (28%) from the baseline to the highest peak during the first set of leg presses. The maximum increase of SBP was 47 mmHg (39%) measured at the peak of the third set.

In the third set, 9 of the AAS patients (38%) had a peak SBP >170 mmHg. Only 2 of them had a peak SBP >180, and none of the AAS patients exceeded 200 mmHg during intervention. The highest measured SBP in the case group was 190 mmHg, and only one patient increased the SBP to this level.

In all three sets of leg presses, HR and SBP increased in the last third of each set and decreased immediately in the 60 sec break in between sets. HR and BP decreased but did not reach baseline values, whereas every new set was started with a higher SBP than previous sets and the peak SBP increased during the 3 sets (pressure load summation).

### 3.2. Comparison to Healthy Controls

**Figure 4:**
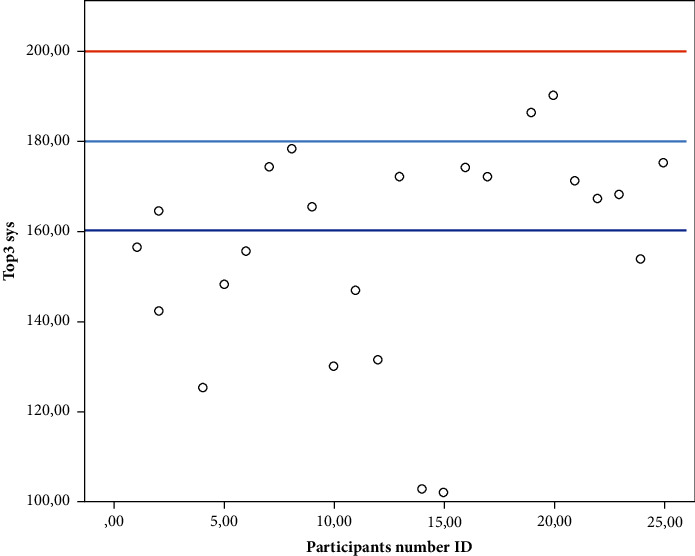
Peak SBP measured in the third set of the leg-press exercise at all the AAS participants. *Y*-axis: Peak SBP (mmHg) of all the AAS participants in the third set. *X*-axis: participants. The red line indicates the pragmatically safety set at 200 mmHg, the light blue line indicates 180 mmHg, and the dark blue line indicates 160 mmHg.

The median peak SBP in the control group increased by 50 mmHg (42%) from the baseline to the peak of the first set of leg presses compared to the 34 mmHg (28%) in the AAS group, and the maximum increase of SBP in the peak of the third set was 58 mmHg (51%) compared to the 47 mmHg (39%) of the AAS patients (Figure 4).

**Figure 5:**
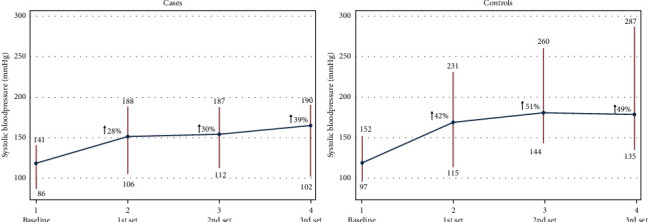
The systolic blood pressure range and the percentage increase from the baseline to the *3*^rd^ set. The systolic blood pressure range and the percentage increase from the baseline to the 1^st^ set, from the baseline to the 2^nd^ set, and from the baseline to the 3^rd^ set.

There was a statistically significant difference in peak SBP between the two groups in all the three sets of leg presses. Five participants (20%) from the control group had a peak SBP >200 mmHg, and no one in the AAS group exceeded 200 mmHg. The mean peak SBP in the case group was significantly different from 200 mmHg (95% CI: −53.5; 33.3, *P* < 0.001) (Figure 4) (Figure 5).

The mean resting heart rate was significantly higher in the AAS group than in the control group. The AAS group had, as expected, a significantly lower resting SBP compared to the control group, probably as a result of the medication use. No difference was seen in the resting DBP.

## 4. Discussion

The present study investigated peak systolic BP in AAS patients during moderate intensity resistance training (60% of maximum strength). The median peak SBP in AAS patients in this study had a maximum increase of 47 mmHg (39%) in the third set of leg presses from the baseline. The highest SBP measured in the AAS group was 190 mmHg in one patient. Compared to the healthy control group, the median peak SBP did not reach the same high level in the AAS patients.

Our findings showed that the AAS patients in control of their blood pressure in this study did not exceed 200 mmHg SBP during moderate intensity resistance training. A new and conservative guideline from the Danish Society of Cardiology on VO_2_ peak [31] tests was published after this study was conducted. The recommendation concerning AAS patients is to avoid SBP >160 mmHg during VO_2_ peak tests. Since the study was conducted before the recommendations were published, blood pressures above 160 mmHg were accepted.

Using this more conservative approach, with an upper limit of 160 mmHg, some of the AAS patients in this study would exceed the limit when performing resistance training at moderate intensity (Figure 3). In this present study, 13 in the AAS group (54%) exceeded 160 mmHg doing the three sets of leg presses at moderate intensity. The mean peak SBP in the AAS group was significantly different from a maximum of 200 mmHg (95% CI: −53.5; 33.3, *p* < 0.001) but not from 160 mmHg (95% CI: −13.5; 6.7, *p*=0.490).

If the AAS patients should follow the recommendation to avoid SBP >160 mmHg, their blood pressure should be monitored during resistance training. Longer breaks are needed between the sets because of the pressure load summation and perhaps lower resistance than 15 RPM could be recommended as well.

Corone et al. found that 75% of their participants had a SBP between 150 and 170 mmHg and the rest had a SBP <150 mmHg during cycling at moderate intensity [32]. In this present study, 54% exceeded 160 mmHg which indicates that maximum SBP during resistance training at moderate intensity in this patient group is comparable with the SBP during cycling at moderate intensity.

Fuglsang et al. measured maximum SBP during the CPET test in 29 AAS patients and documented a mean of 207 ± 33 mmHg [21]. This is much higher than the highest mean peak in the third set at 153 mmHg ± 23 in this present study. Only one participant reached 190 mmHg.

This illustrates (based on a relatively small sample size) that it is possible for this patient group to stay below 200 mmHg when performing three sets of resistance training at moderate intensity with 15 RM.

AAS patients are instructed to lift nothing heavier than they are able to breathe normally during the lift in order to avoid Valsalva Maneuver and unfavorable high SBP increases [7, 18, 20]. Several studies have shown that if participants performed Valsalva Maneuver during lifting, they would increase the risk of peak SBP >200 mmHg [23, 29, 30]. That is why all participants were instructed to breathe the same way during the leg presses, which was to exhale during the most strenuous phase of the repetition and inhale during the less strenuous phase of the repetition.

The continuous monitoring of the BP with the Nexfin monitor was very useful in detecting unfavorable high increases in SBP during resistance training for this patient group. The monitor showed BP at a given time, and during the intervention, an instructor was noting the highest measurements in all sets of leg presses. The noted values matched the values in the beat-to-beat data.

Chaddha et al. [8] highlighted fear as the limiting factor for physical activity after aortic dissection. We sensed a fear of lifting heavy in some of our participants in the case group, maybe because they feared that unfavorable high increases in SBP could cause them harm. This could be a reason to use the Nexfin monitor for pedagogical interventions for AAS patients doing resistance training as this group of patients may have a fear of SBP increasing rapidly during physically activity [8].

Although invasive measurements of BP are superior compared to the noninvasive methodology in terms of sensitivity, the noninvasive approach is acceptable for clinical rehabilitation purposes as demonstrated in several studies [25–27]. The continuous monitoring of BP using a noninvasive finger arterial clamp method may be useful in detecting an unfavorable high increase in BP during resistance training.

Using the Nexfin monitor in training sessions following AAS may have a positive influence on the fear of exercise and ability and motivation to perform resistance training. This hypothesis needs to be further investigated.

There are certain limitations to this study. Though the Nexfin monitor was useful, it had some limitations, e.g., Schattenkerk et al. and Imholz et al. [24, 26, 29] published data indicating that the Nexfin monitor tends to overestimate systolic and underestimate diastolic pressure compared to traditional BP monitors. With this knowledge and no SBP measurements >200 mmHg in the AAS group, none of our participants were exposed to any danger concerning their SBP increases. A limitation with the arterial clamp was the sensitivity. In this current study, a couple of the participants had arthritis or cold fingers and the finger clamps were changed several times, moved to another finger, and to the other hand, until able to measure a valid SBP. A limit to our protocol was the continuous repetitions of sets with only 60 seconds of breaks in between each set [29]. This might have had an influence in higher peak SBP in the leg presses. SBP of the participants did not decrease to the baseline during the 60 second break between each set.

## 5. Conclusion

In conclusion, our study indicates that AAS patients in control of their BP endure to perform resistance training at moderate intensity (60% of maximum strength). The resistance training examined in this study does not lead to SBP in excess of 200 mmHg if a maximum of three sets are performed including a break of 60 seconds in between every set. However, to avoid SBP >160 mmHg, there is a need of longer breaks between the sets and the intensity of resistance training that should be lowered to 40–50% of maximum strength.

The results are based on a small sample and are limited to AAS patients in control of their SBP, and further investigations regarding safety and intensity levels are needed.

## Figures and Tables

**Table 1:** Baseline characteristics of the participants.

	Cases (*n* = 24)	Healthy controls (*n* = 24)	Mean difference	95% confidence intervals	*p* value
Sex	10 f/14 m	10 f/14 m			

*Age (years)*					
Mean ± SD	60.5 ± 11.9	60.2 ± 11.8	0.3	(−6.6; 7.2)	*p*=0.934
Range	39.0–75.8	36.75–76.2			

*Weight (kg)*					
Mean ± SD	83.5 ± 24.2	71.2 ± 11.9	12.3	(1.2; 23.4)	*p*=0.037
Range	55–162	51–96			

*Height (cm)*					
Mean ± SD	175.8 ± 12.6	174.3 ± 8.5	1.5	(−4.8; 7.7)	*p*=0.894
Range	150–194	158–190			

*BMI (kg/m* ^*2*^)					
Mean ± SD	26.6 ± 5.6	23.5 ± 2.8	3.1	(0.5; 5.7)	*p*=0.021
Range	20.3–46.3	19–30.5			

*Resting HR*					
Mean ± SD	71.1 ± 13.8	61.7 ± 10.2	9.5	(2.4; 16.5)	*p*=0.010
Range	53–106	42–79			

*Resting SBP (mmHg)*					
Mean ± SD	120.6 ± 12.9	128.0 ± 11.8	−7.5	(−14.6; −0.3)	*p*=0.042
Range	96–145	100–148			

*Resting DBP (mmHg)*					
Mean ± SD	71.7 ± 9.7	75.9 ± 8.5	−4.2	(−9.5; 1.1)	*p*=0.119
Range	51–94	59–89			
Antihypertensive	8/	3/			
Treatment	6/	1^*∗*^/			
1 drug/2 drugs/3	4/	0/			
Drugs/4 drugs	4	0			

*Etiology*:					
Aneurysms or dilatations/	17/				
	3/				
Marfan syndrome/dissection	4				

*n*: numbers, f: female, m: male, SD: standard deviation, BMI: body mass index, HR: heart rate, SBP: systolic blood pressure, DBP: diastolic blood pressure, and ^*∗*^: lowest possible dose of medication.

**Table 2:** Hemodynamic outcomes and workload in the two groups during resistance training at moderate intensity.

		Baseline	1st set	2nd set	3rd set
Cases	*SBP (mmHg)*				
	Mean ± SD	115.7 ± 13.5	148.6 ± 19.9	153.3 ± 22.8	156.6 ± 23.9
	Median	118.6	152.1	154.1	165.2
	Range	86.0–140.8	105.8–188.0	112.4–187.2	102.0–190.4
	*DBP (mmHg)*				
	Mean ± SD	74.3 ± 9.6	93.2 ± 14.9	95.5 ± 13.1	96.6 ± 13.5
	Median	73.6	91.6	98.0	96.8
	Range	56.0–97.0	68.6–117.43	69.6–129.13	69.0–134.70
	*HR (bpm)*				
	Mean ± SD	88.8 ± 13.2	106.9 ± 19.8	109.4 ± 18.9	108.9 ± 20.8
	Median	87.1	101.9	102.4	102.8
	Range	69.2–116.0	81.6–165.4	87.6–159.3	81.4–160.6
	*Resistance (kg)*				
	Mean ± SD	117.9 ± 40.1			
	Median	130			
	Range	40–200			

Healthy controls	*SBP (mmHg)*				
	Mean ± SD	121.6 ± 13.5	163.6 ± 28.7	177.8 ± 37.5	185.5 ± 32.3
	Median	120.1	170.1	181.3	179.3
	Range	97.3–152.8	114.6–231.2	143.5–260.2	135.2–286.7
	*DBP (mmHg)*				
	Mean ± SD	74.4 ± 9.3	100.2 + 15.0	111.4 + 18.6	109.5 + 17.2
	Median	73.2	98.6	106.3	107.3
	Range	60.4–97.1	80.3–133.3	85.0–162.4	73.8–149.5
	*HR (bpm)*				
	Mean ± SD	80.5 ± 16.9	100.0 ± 14.4	110.7 ± 18.1	108.1 ± 17.1
	Median	80.5	106.9	108.2	109.4
	Range	51.7–112.3	85.8–128.0	78.3–131.8	84.4–142.1
	*Resistance (kg)*				
	Mean ± SD	136.7 ± 43.8			
	Median	130			
	Range	70–230			

Mean difference between cases and healthy controls	SBP (mmHg) (95% CI)	−5.9 (−13.6; 1.9) *p*=0.135	−15.1 (−29.4; −0.7) *p*=0.040	−27.1 (−46.4; −7.8) *p*=0.007	−28.9 (−45.4; −12.4) *p*=0.001
	DBP (mmHg) (95% CI)	0.1 (−5.6; 5.4) *p*=0.978	−6.8 (−15.9; 1.7) *p*=0.115	−15.9 (−25.8; −6.0) *p*=0.002	−11.5 (−20.4; −2.5) *p*=0.013
	HR (bpm) (95% CI)	8.3 (−0.5; 17.1) *p*=0.064	−2.6 (−8.9; 10.0) *p*=0.905	−1.1 (−10.1; 8.1) *p*=0.820	−0.7 (−11.0; 9.6) *p*=0.891
	Resistance (kg) (95% CI)	−18.8 (−43.2; 5.7) *p*=0.129			

SBP: systolic blood pressure, SD: standard deviation, DBP: diastolic blood pressure, HR: heart rate, bpm: beat per minute, and 95% CI: 95% confidence intervals. All hemodynamic outcomes were measured using the Nexfin device.

## Data Availability

The data used to support the findings of this study are available from the corresponding author upon request.
